# Proteomic profiling of isolated mouse endometrial epithelial cells reveals coordinated redox and endoplasmic reticulum stress-associated pathways during uterine receptivity

**DOI:** 10.14202/vetworld.2026.2734-2750

**Published:** 2026-07-06

**Authors:** Jakree Jitjumnong, Wilasinee Inyawilert, Attapol Tiantong, Shih-Han Wang, Chao-Jung Chen, Yu-Jing Liao, Tossapol Moonmanee, San-Yuan Huang, Pin-Chi Tang

**Affiliations:** 1 Department of Animal and Aquatic Sciences, Faculty of Agriculture, Chiang Mai University, Chiang Mai 50200, Thailand.; 2 Functional Feed Innovation Center, Faculty of Agriculture, Chiang Mai University, Chiang Mai 50200, Thailand.; 3 Center of Omics for High-Value Agriculture (AgOmics-CMU), Faculty of Agriculture, Chiang Mai University, Chiang Mai 50200, Thailand.; 4 Department of Agricultural Science, Faculty of Agriculture, Natural Resources and Environment, Naresuan University, Phitsanulok 65000, Thailand.; 5 The Center of Agricultural Biotechnology, Naresuan University, Phitsanulok 65000, Thailand.; 6 Faculty of Animal Sciences and Agricultural Technology, Silpakorn University, Phetchaburi IT Campus, Cha-Am, Phetchaburi 76120, Thailand.; 7 Institute of Biomedical Sciences, Academia Sinica, Taipei 115, Taiwan.; 8 Proteomic Core Laboratory, Department of Medical Research, China Medical University Hospital, Taichung 40447, Taiwan.; 9 Graduate Institute of Integrated Medicine, China Medical University, Taichung 40402, Taiwan.; 10 Genetics and Physiology Division, Taiwan Livestock Research Institute, Ministry of Agriculture, Tainan 71246, Taiwan.; 11 Department of Animal Science, National Chung Hsing University, Taichung 40227, Taiwan.; 12 The iEGG and Animal Biotechnology Research Center, National Chung Hsing University, Taichung 40227, Taiwan.; 13 Professional Master Program of Agricultural Business Management, National Chung Hsing University, Taichung 40227, Taiwan.

**Keywords:** endometrial receptivity, endoplasmic reticulum stress, gstm2, implantation, mouse uterus, proteomics, redox regulation, uterine epithelium

## Abstract

**Background and Aim:**

Uterine receptivity is critical for successful embryo implantation, yet epithelial-specific proteomic changes during this transition remain incompletely characterized. This study aimed to profile changes in protein expression in isolated mouse endometrial epithelial cells between the pre-receptive (Day 1) and receptive (Day 4) phases of pregnancy to identify key pathways associated with uterine receptivity.

**Materials and Methods:**

Endometrial epithelial cells were isolated from pregnant CD-1 mice on Days 1 and 4 of pregnancy. Protein extracts were analyzed using two-dimensional gel electrophoresis followed by matrix-assisted laser desorption/ionization time-of-flight mass spectrometry for identification. Differentially expressed proteins were functionally annotated using Gene Ontology. Key candidates, including Gstm2 and vimentin, were validated by immunofluorescence and quantitative real-time polymerase chain reaction.

**Results:**

Approximately 674 protein spots were detected, of which 80 were differentially expressed (p < 0.05, ≥2-fold change) and 52 were successfully identified. These proteins were primarily associated with cellular metabolism, redox regulation, protein folding, and endoplasmic reticulum (ER) stress responses. Coordinated upregulation of antioxidant proteins (Gstm2, Gstm7, Prdx2, Cat) and ER stress-associated proteins (PDIA3, HSPA5) was observed on Day 4. Gstm2 showed consistent upregulation at both protein and transcript levels (p < 0.05) with enhanced epithelial localization, while vimentin expression remained stable, supporting cytoskeletal readiness. These changes indicate integrated redox homeostasis and stress adaptation during the acquisition of uterine receptivity.

**Conclusion:**

This epithelial-specific proteomic analysis reveals coordinated redox and ER stress-associated pathways during the transition to uterine receptivity in mice. The findings provide a focused proteome resource that highlights Gstm2 as a candidate regulator within antioxidant networks and establishes a foundation for understanding molecular mechanisms at the embryo–maternal interface.

## INTRODUCTION

Successful embryo implantation requires the coordinated progression of blastocyst development and synchronized adaptation of the uterine endometrium. The endometrium plays a central role in this process, and inadequate uterine preparation can result in implantation failure [1–3]. Throughout the estrous cycle, the endometrium undergoes dynamic structural and functional modifications to support embryo implantation [1–3]. These changes are tightly regulated by ovarian steroid hormones, cytokines, and growth factors, which collectively influence endometrial differentiation, pregnancy recognition signaling, uterine receptivity, and embryo–maternal interactions [1, 4, 5]. Embryo implantation is a highly coordinated process involving reciprocal communication between the blastocyst and the uterine epithelium [1, 3]. In rodents, trophoblast giant cells play a critical role in decidual invasion and placental formation [1, 6, 7], while cytoskeletal remodeling is essential for trophoblast differentiation and tissue reorganization during implantation [8–10].

In recent years, proteomic approaches have increasingly enabled the identification of dynamic protein-level changes associated with endometrial receptivity and implantation success [11, 12]. Advances in mass spectrometry-based proteomics, together with multi-omics integration, have enhanced our understanding of protein expression dynamics during the implantation window [11, 12]. Previous transcriptomic and multi-omics studies have provided important insights into uterine receptivity, primarily at the whole-tissue level, capturing combined signals from epithelial, stromal, and immune compartments [3, 11, 12]. However, cell type–specific proteomic characterization of the endometrial epithelium remains limited. Given that the luminal epithelium represents the primary interface for embryo–maternal interaction, targeted analysis of epithelial cells is essential to better define localized molecular changes associated with implantation. Whole-tissue analyses inherently reflect composite signals from epithelial, stromal, and immune compartments, which may obscure cell type–specific molecular changes critical for implantation [11, 12]. In contrast, the luminal epithelium serves as the first point of contact between the blastocyst and the maternal environment, mediating attachment, luminal closure, and early embryo–maternal signaling [3, 13]. Therefore, epithelial-specific proteomic analysis provides a more precise framework to resolve localized molecular adaptations at the implantation interface that may not be captured by whole-tissue approaches. Moreover, emerging evidence highlights redox regulation and antioxidant defense as key modulators of uterine receptivity [14–16]. Progesterone-mediated signaling and prostaglandin pathways also play crucial roles in regulating endometrial remodeling and implantation competence [1, 4, 17].

Despite these advances, significant knowledge gaps remain regarding the molecular mechanisms that govern epithelial adaptation during the establishment of uterine receptivity. Most previous investigations have focused on transcriptomic profiling or whole-endometrial tissue analyses, which do not adequately distinguish epithelial-specific responses from those occurring in stromal, vascular, or immune cell populations [3, 11, 12]. Consequently, the protein-level regulatory networks operating specifically within endometrial epithelial cells during the peri-implantation period remain insufficiently characterized. In particular, the coordinated involvement of redox homeostasis, endoplasmic reticulum stress responses, protein folding pathways, and cytoskeletal remodeling in facilitating epithelial receptivity has not been comprehensively explored. Because proteins represent the primary functional mediators of cellular activity, epithelial-specific proteomic profiling may provide critical insights that cannot be inferred solely from transcriptomic datasets. Addressing this gap is essential for improving our understanding of the molecular events that support embryo attachment and implantation and may contribute to the identification of biomarkers or regulatory pathways associated with reproductive success.

Therefore, this study aimed to characterize epithelial-specific proteomic changes in mouse endometrial cells during early pregnancy and to identify candidate regulators associated with uterine receptivity. By combining two-dimensional gel electrophoresis-based proteomic analysis with protein identification and molecular validation, this study sought to establish a focused proteomic resource for the receptive endometrial epithelium. Particular emphasis was placed on identifying proteins and pathways associated with redox regulation, endoplasmic reticulum (ER) stress adaptation, and epithelial remodeling during the transition from the pre-receptive to receptive uterine state.

## MATERIALS AND METHODS

### Ethical approval

All procedures involving animals were reviewed and approved by the Institutional Animal Care and Use Committee of National Chung Hsing University, Taichung, Taiwan, under permit number 99-83. The study was conducted in accordance with institutional animal welfare regulations, relevant international guidelines for laboratory animal care and use, and the ARRIVE 2.0 guidelines. All efforts were made to minimize animal suffering and reduce the number of animals used, in accordance with the principles of Replacement, Reduction, and Refinement. Animals were maintained under controlled housing conditions, acclimatized before experimentation, and handled by trained personnel. Sample collection was performed using standardized procedures, and biological pooling was applied to obtain sufficient material for proteomic analysis while limiting unnecessary animal use.

### Study period and location

The study was conducted in the Department of Animal Science and the iEGG and Animal Biotechnology Research Center, National Chung Hsing University, Taichung, Taiwan. Animal housing, sample collection, protein extraction, proteomic analysis, immunofluorescence analysis, RNA isolation, quantitative real-time polymerase chain reaction (qPCR), and statistical analysis were performed under controlled laboratory conditions at the institution.

### Study design

Sexually mature female CD-1 mice (8 weeks old, 30–35 g, n = 18) were purchased from BioLASCO Taiwan, Taipei, Taiwan. Mice were housed in individually ventilated cages under specific pathogen-free conditions with controlled temperature (22 ± 2°C), relative humidity (50%–60%), and a 14:10 h light-dark cycle. Animals had ad libitum access to standard rodent chow and water and were acclimatized for at least 7 days before experimentation. Mice were randomly assigned to mating pairs. Estrous cycle staging was performed by vaginal cytology before mating to ensure synchronization. Females were housed overnight with fertile males of the same strain, and successful mating was confirmed the following morning by the presence of a vaginal plug, designated as Day 1 of pregnancy. Animals were then allocated into two experimental groups corresponding to Day 1 and Day 4 of pregnancy (n = 9 per group). Chemicals were purchased from Sigma-Aldrich (St. Louis, MO, USA).

### Sample collection

Pregnancy was confirmed by the presence of a vaginal plug (Day 1), and Day 4 samples were collected at the expected peri-implantation stage based on established mouse reproductive timelines; however, direct confirmation of blastocyst presence by uterine flushing was not performed. One biological replicate consisted of pooled endometrial epithelial cells collected from three mice on either Day 1 or Day 4 of gestation. Three independent biological replicates were included for each group (total n = 9 mice per group). Pooling was performed to ensure sufficient protein yield for 2-DE analysis and to reduce inter-individual variability inherent in biological samples. Endometrial isolation was performed as previously described [18]. Although epithelial cells were isolated using an established protocol, no additional quantitative assessment of epithelial purity was performed. Therefore, minor contamination from stromal cells cannot be completely excluded. Briefly, excised uterine horns were flushed with 0.75% ethylenediaminetetraacetic acid (EDTA; pH 7.4) in Dulbecco's phosphate-buffered saline and incubated at 37°C for 20 min under 5% CO₂. The endometrial epithelium was then carefully separated from the inner uterine wall for protein extraction. Endometrial epithelial cells were lysed in extraction buffer containing 9.5 M urea, 2% NP-40, 2% (v/v) pharmalyte (pH 3–10), and 65 mM dithiothreitol (DTT), supplemented with a protease inhibitor cocktail. Lysates were stored at −80°C until 2-DE analysis. Protease inhibitor cocktail (Roche Complete™, Roche Diagnostics, Basel, Switzerland, EDTA-free) was added fresh at 1× concentration. Protein concentration was determined using the Bradford assay (Bio-Rad Protein Assay, Bio-Rad Laboratories, Hercules, CA, USA) with bovine serum albumin as the standard.

### Two-dimensional gel electrophoresis

Total protein concentration was determined using the Bradford assay according to the manufacturer's instructions before electrophoresis. Equal amounts of protein (400 µg) were loaded for each biological replicate to ensure consistency across samples. Soluble proteins were extracted from pooled endometrial epithelial tissues collected on Days 1 and 4 of pregnancy, with three independent biological replicates per group. For 2-DE, 400 µg of total protein per sample was applied to 18-cm immobilized pH gradient (IPG) strips (pH 3–10 linear; Amersham Biosciences, Uppsala, Sweden) using an IPGphor system (Amersham Biosciences, Uppsala, Sweden). Protein samples were mixed with 175 µL of IEF buffer (9.5 M urea, 2% NP-40, 2% pharmalyte 3–10, and 65 mM dithiothreitol [DTT]), followed by an equal volume of rehydration buffer (8 M urea, 2% CHAPS, and 0.5% pharmalyte 3–10) to a final volume of 350 µL. Samples were loaded onto strip holders and rehydrated at 30 V for 12 h. Isoelectric focusing (IEF) was performed at 20°C with a voltage gradient: 500 V for 1 h, 1000 V for 1 h, then 8000 V over 3 h, followed by 8000 V until 64,000 Vh total. Second-dimension gels were run at 15°C with constant power of 2.5 W/gel for 25 min, then 6 W/gel until the bromophenol blue front reached the bottom of the gel (approximately 7–8 h). Gels were stained overnight with colloidal Coomassie Brilliant Blue (Serva Electrophoresis, Heidelberg, Germany), neutralized with 0.1 M Tris-phosphoric acid (pH 6.5), destained with 25% methanol, and washed with distilled water. Gel images were acquired using a laser densitometer and processed with ImageQuant software. No exogenous internal standard was included for 2-DE; therefore, protein expression levels were normalized using the relative volume (RVol) method in Melanie 7 software, in which each spot's volume was normalized to the total volume of all detected spots on the same gel. All samples were processed under identical experimental conditions, and gel images were assessed for quality and reproducibility across biological replicates. Only gels showing consistent spot patterns and without evident artifacts were included for downstream analysis. Experimental procedures and reporting were conducted in accordance with established best practices for gel-based proteomics to ensure methodological transparency and reproducibility. This study complies with the Minimum Information About a Proteomics Experiment guidelines for gel-based proteomics.

### Analysis of differential protein expression

Spot detection and matching were performed automatically using Melanie 7 software (version 7; GeneBio, Geneva, Switzerland), followed by manual verification. Although full blinding was not implemented, automated image analysis and standardized processing were applied to minimize potential bias. All samples were processed and analyzed under identical experimental conditions to ensure consistency. Although analyses were not performed in a fully blinded manner due to the nature of gel-based proteomics workflows, automated image analysis and standardized processing were applied to minimize potential bias. A total of 674 protein spots were detected across all gels. To normalize staining variability, the RVol of each spot was calculated as the ratio of the spot volume to the total volume of all detected spots. Differential expression was determined based on the ratio of RVol values between groups [19, 20]. For interpretation, a ratio (Day 1/Day 4) >1 indicates higher expression on Day 1 (downregulated on Day 4), whereas a ratio <1 indicates higher expression on Day 4 (upregulated on Day 4).

### Protein identification

Protein spots showing significant differential expression between Days 1 and 4 were excised and subjected to in-gel digestion following previously described protocols with minor modifications. Excised gel spots were washed with double-distilled water, followed by 50% acetonitrile in 50 mM ammonium bicarbonate and then pure acetonitrile, and subsequently dried in a SpeedVac concentrator. Excised gel spots were destained, reduced with 10 mM dithiothreitol, alkylated with 55 mM iodoacetamide, and digested with sequencing-grade trypsin (20 ng/µL; Promega, Madison, WI, USA) overnight at 37°C. Peptides were extracted using acetonitrile/trifluoroacetic acid solution, followed by sonication, and subjected to MALDI-TOF/MS analysis. MS and MS/MS data were processed using the Mascot search engine against the National Center for Biotechnology Information non-redundant database with taxonomy restricted to *Mus musculus*. Carbamidomethylation (C) was set as a fixed modification, and oxidation (M) as a variable modification. Trypsin was specified as the digestion enzyme with one missed cleavage allowed. The peptide mass tolerance was set to ±100 ppm and fragment mass tolerance to ±0.5 Da. Protein identification was considered significant when Mascot scores exceeded the p < 0.05 threshold. Functional annotation of the identified proteins was performed using the Gene Ontology database (AmiGO 2). MALDI-TOF/MS analysis was performed using a Bruker Autoflex III mass spectrometer (Bruker Daltonics, Bremen, Germany) operated in reflector positive-ion mode. Spectra were acquired by accumulating 200–500 laser shots per spot. External calibration was performed using Bruker Peptide Calibration Standard II. Peak detection and processing were carried out using FlexAnalysis software with a signal-to-noise ratio threshold >3 and a minimum resolution of 500.

### Immunofluorescence analysis

Immunofluorescence staining was performed on 5-μm paraffin-embedded uterine sections collected on Days 1 and 4 of pregnancy. Sections were mounted on poly-L-lysine-coated slides, dried overnight at 37°C, and incubated at 65°C for 10 min before deparaffinization in xylene and rehydration through a graded ethanol series. Antigen retrieval was carried out in 0.01 M sodium citrate buffer (pH 6.0) at 98°C for 20 min. Sections were washed in Tris-buffered saline containing 0.05% Tween-20 (TBST) and blocked with 3% bovine serum albumin in TBST for 1 h 25°C ± 25°C to reduce nonspecific binding. Sections were incubated overnight at 4°C with primary antibodies against *Gstm2* (1:200; PA5-75995; Invitrogen, Carlsbad, CA, USA), vimentin (1:200; PA5-27231; Invitrogen), and PCNA (1:200; PA5-27214; Invitrogen). All primary antibodies were commercially obtained and used according to the manufacturer's instructions. Antibody specificity was evaluated using negative controls (omission of primary antibody), which showed no detectable signal, and by confirming that staining patterns were consistent with previously reported localization in uterine tissues. Following primary antibody incubation, sections were washed and incubated with fluorescein isothiocyanate (FITC)-conjugated anti-rabbit secondary antibody (1:200; ab150077; Abcam, Cambridge, UK) for 1 h at 37°C in the dark. Nuclei were counterstained with DAPI. Fluorescence images were captured using a fluorescence microscope with identical exposure settings across all groups to enable qualitative comparison. Image acquisition parameters were kept constant across samples to ensure consistency in signal interpretation.

### RNA isolation and qPCR

Total RNA was extracted from endometrial epithelial cells using TRIzol reagent (Invitrogen, Carlsbad, CA, USA) according to the manufacturer's instructions. RNA quality was assessed by NanoDrop spectrophotometry (Thermo Fisher Scientific, Wilmington, DE, USA; A260/280 >1.8, A260/230 >2.0) and agarose gel electrophoresis. cDNA was synthesized from 1 µg total RNA. RNA was reverse-transcribed into complementary DNA using the Transcriptor First Strand cDNA Synthesis Kit (Roche Diagnostics, Basel, Switzerland). qPCR was performed using a StepOne Real-Time PCR System (Applied Biosystems, Foster City, CA, USA). Relative gene expression levels of vimentin and *Gstm2* were calculated using the comparative Ct (2^−ΔΔCt) method, with glyceraldehyde-3-phosphate dehydrogenase as the internal control. Primer specificity was verified by melt curve analysis, which showed a single peak for each target gene, and by agarose gel electrophoresis, which showed a single product of the expected size. Primer efficiencies (90%–110%) were determined using standard curve analysis. Melt curve analysis confirmed the amplification of a single specific product for each gene. No-template controls were included to verify the absence of contamination. Primer sequences and amplicon sizes are provided in Table 1.

**Table 1:** Primer sequences used for quantitative real-time polymerase chain reaction.

**Gene**	**Forward primer (5** **′–** **3** **′** **)**	**Reverse primer (5** **′–** **3** **′** **)**	**Product size (bp)**	**Accession No.**
*GAPDH*	GTCGTGGAGTCTACTGGTGTC	GAGCCCTTCCACAATGCCAAA	240	NM_001357943.2
*Vimentin*	CGGCTGCGAGAGAAATTGC	CCACTTTCCGTTCAAGGTCAAG	124	NM_011701.4
*Gstm2*	CCATGGTTTGCAGGGAACAAG	AGAAGAAAGCTGCACGTGGT	300	NM_008183.4

### Statistical analysis

Data are presented as mean ± standard error of the mean. Differences between Day 1 and Day 4 groups were analyzed using a two-tailed unpaired Student's t-test (SAS version 9.1; SAS Institute Inc., Cary, NC, USA), with p < 0.05 considered statistically significant. For proteomic analysis, proteins were considered differentially expressed based on combined criteria of p < 0.05 and ≥2-fold change. GraphPad Prism 9 (GraphPad Software, San Diego, CA, USA) was used to visualize qPCR and immunofluorescence data. Given the exploratory nature of two-dimensional gel-based proteomics, this combined threshold was applied to reduce potential false-positive findings. With 674 protein spots analyzed, approximately 5% (≈34 spots) would be expected to be identified as significant by chance alone at this threshold. Although a formal false discovery rate correction was not applied, selected candidate proteins were independently validated by immunofluorescence and quantitative real-time PCR, providing additional biological support for the observed changes. Future studies employing high-resolution quantitative proteomics with appropriate multiple-testing correction are warranted to further enhance statistical robustness. No formal power analysis was conducted, as this study was designed as an exploratory proteomic investigation. Instead, stringent selection criteria combined with independent validation experiments were applied to enhance the reliability of the findings.

## RESULTS

### Profile of mouse endometrial epithelial proteome during early pregnancy

**Figure 1:**
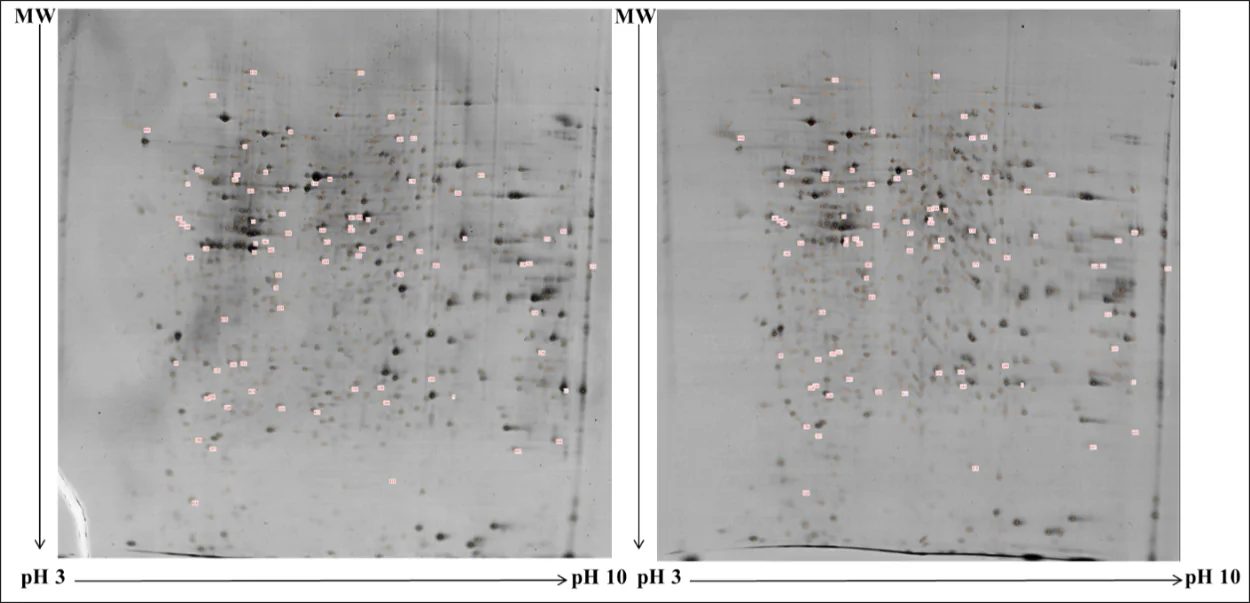
Representative two-dimensional gels depicting the proteome profiles of mouse endometrial epithelial cells on Days 1 and 4 of pregnancy.

Endometrial epithelial cells were collected from mice on Days 1 and 4 of pregnancy to examine protein expression using two-dimensional gel electrophoresis (2-DE), followed by colloidal Coomassie Brilliant Blue staining. Figure 1 shows a representative 2-DE reference map of the mouse endometrial epithelial proteome generated using pH 3–10 immobilized pH gradient strips and 10% SDS-polyacrylamide gels. Approximately 674 distinct protein spots were detected across samples collected on Days 1 and 4 of pregnancy.

### Quantitative changes in endometrial protein expression from Day 1 to Day 4 of pregnancy

To compare protein expression profiles between Days 1 and 4 of pregnancy, 2-DE gel images were analyzed using Melanie software (version 7). A total of 674 protein spots were consistently detected across all gels. Among these, 80 protein spots exhibited significant differential expression between the two time points (p < 0.05 and ≥2.0-fold change). The list of differentially expressed proteins is provided in Table 2. Of these, 42 proteins were upregulated on Day 4 (ratio <1), whereas 38 proteins were downregulated on Day 4 (ratio >1) relative to Day 1. Subsequent identification by MALDI-TOF/MS and MALDI-TOF/TOF analysis successfully characterized 52 of the 80 differentially expressed protein spots (Table 3). Importantly, this dataset represents a cell type–specific proteomic resource derived from isolated endometrial epithelial cells, providing a focused reference for molecular changes occurring at the embryo–maternal interface during the transition to uterine receptivity.

**Table 2:** Differentially expressed proteins in mouse endometrial cells during early pregnancy.

Spot No.	Day 1 volume (%)	Day 4 volume (%)	Ratio (Day 1/Day 4)	Regulation (Day 4 vs Day 1)	p-value
**7**	0.10 ± 0.04ᵇ	0.52 ± 0.05ᵃ	0.19	Upregulated on Day 4	0.000283
**11**	0.76 ± 0.19ᵃ	0.22 ± 0.18ᵇ	3.45	Downregulated on Day 4	0.023193
**16**	0.54 ± 0.12ᵃ	0.27 ± 0.03ᵇ	2.00	Downregulated on Day 4	0.019474
**23**	1.21 ± 0.24ᵃ	0.58 ± 0.13ᵇ	2.08	Downregulated on Day 4	0.016044
**29**	1.12 ± 0.24ᵃ	0.43 ± 0.16ᵇ	2.60	Downregulated on Day 4	0.014381
**33**	0.10 ± 0.02ᵇ	0.23 ± 0.07ᵃ	0.43	Upregulated on Day 4	0.040713
**39**	0.21 ± 0.02ᵃ	0.10 ± 0.009ᵇ	2.10	Downregulated on Day 4	0.010155
**46**	0.32 ± 0.05ᵃ	0.15 ± 0.08ᵇ	2.13	Downregulated on Day 4	0.000891
**52**	0.13 ± 0.02ᵃ	0.06 ± 0.02ᵇ	2.17	Downregulated on Day 4	0.008505
**55**	0.19 ± 0.01ᵇ	0.39 ± 0.04ᵃ	0.49	Upregulated on Day 4	0.025876
**56**	0.09 ± 0.04ᵇ	0.28 ± 0.05ᵃ	0.32	Upregulated on Day 4	0.004535
**57**	0.09 ± 0.02ᵇ	0.18 ± 0.04ᵃ	0.50	Upregulated on Day 4	0.005394
**60**	0.05 ± 0.01ᵇ	0.12 ± 0.02ᵃ	0.42	Upregulated on Day 4	0.022701
**67**	0.05 ± 0.007ᵇ	0.17 ± 0.04ᵃ	0.29	Upregulated on Day 4	0.015278
**72**	0.41 ± 0.04ᵃ	0.20 ± 0.09ᵇ	2.05	Downregulated on Day 4	0.009048
**76**	0.12 ± 0.03ᵇ	0.29 ± 0.06ᵃ	0.41	Upregulated on Day 4	0.032291
**94**	0.21 ± 0.05ᵃ	0.07 ± 0.03ᵇ	3.00	Downregulated on Day 4	0.014791
**100**	0.08 ± 0.02ᵃ	0.043 ± 0.01ᵇ	1.86	Downregulated on Day 4	0.011585
**116**	0.33 ± 0.12ᵃ	0.05 ± 0.02ᵇ	6.60	Downregulated on Day 4	0.000400
**137**	0.08 ± 0.008ᵃ	0.03 ± 0.01ᵇ	2.67	Downregulated on Day 4	0.000410
**141**	0.02 ± 0.007ᵇ	0.10 ± 0.01ᵃ	0.20	Upregulated on Day 4	0.002816
**144**	0.05 ± 0.01ᵇ	0.21 ± 0.02ᵃ	0.20	Upregulated on Day 4	0.002468
**145**	0.04 ± 0.009ᵇ	0.28 ± 0.06ᵃ	0.14	Upregulated on Day 4	0.011013
**154**	0.16 ± 0.02ᵃ	0.07 ± 0.008ᵇ	2.29	Downregulated on Day 4	0.001079
**156**	0.02 ± 0.02ᵇ	0.09 ± 0.009ᵃ	0.22	Upregulated on Day 4	0.012501
**160**	0.24 ± 0.02ᵃ	0.09 ± 0.02ᵇ	2.67	Downregulated on Day 4	0.023801
**179**	0.20 ± 0.04ᵃ	0.09 ± 0.02ᵇ	2.22	Downregulated on Day 4	0.008831
**197**	0.10 ± 0.03ᵇ	0.30 ± 0.09ᵃ	0.33	Upregulated on Day 4	0.005713
**211**	0.06 ± 0.01ᵇ	0.15 ± 0.03ᵃ	0.40	Upregulated on Day 4	0.003560
**222**	0.16 ± 0.02ᵃ	0.08 ± 0.02ᵇ	2.00	Downregulated on Day 4	0.017929
**224**	0.14 ± 0.02ᵇ	0.28 ± 0.03ᵃ	0.50	Upregulated on Day 4	0.007739
**229**	0.15 ± 0.03ᵃ	0.06 ± 0.03ᵇ	2.50	Downregulated on Day 4	0.000076
**230**	0.17 ± 0.03ᵃ	0.07 ± 0.02ᵇ	2.43	Downregulated on Day 4	0.012954
**236**	0.13 ± 0.006ᵇ	0.33 ± 0.02ᵃ	0.39	Upregulated on Day 4	0.001501
**246**	0.07 ± 0.02ᵇ	0.21 ± 0.05ᵃ	0.33	Upregulated on Day 4	0.028508
**249**	0.06 ± 0.01ᵇ	0.16 ± 0.02ᵃ	0.38	Upregulated on Day 4	0.012756
**254**	0.15 ± 0.04ᵃ	0.07 ± 0.02ᵇ	2.14	Downregulated on Day 4	0.020577
**264**	0.43 ± 0.08ᵃ	0.21 ± 0.03ᵇ	2.05	Downregulated on Day 4	0.001970
**276**	0.22 ± 0.02ᵃ	0.11 ± 0.0ᵇ	2.00	Downregulated on Day 4	0.007829
**278**	0.10 ± 0.04ᵇ	0.27 ± 0.05ᵃ	0.37	Upregulated on Day 4	0.005011
**279**	0.13 ± 0.06ᵇ	0.32 ± 0.007ᵃ	0.41	Upregulated on Day 4	0.025550
**284**	0.13 ± 0.03ᵃ	0.06 ± 0.03ᵇ	2.20	Downregulated on Day 4	0.000004
**285**	0.13 ± 0.02ᵇ	0.51 ± 0.006ᵃ	0.25	Upregulated on Day 4	0.014231
**287**	0.25 ± 0.06ᵃ	0.09 ± 0.02ᵇ	2.78	Downregulated on Day 4	0.028098
**300**	0.10 ± 0.02ᵃ	0.04 ± 0.02ᵇ	2.50	Downregulated on Day 4	0.033515
**302**	0.18 ± 0.06ᵇ	0.36 ± 0.08ᵃ	0.50	Upregulated on Day 4	0.016860
**314**	0.45 ± 0.07ᵃ	0.17 ± 0.10ᵇ	2.65	Downregulated on Day 4	0.013880
**315**	0.42 ± 0.09ᵃ	0.13 ± 0.08ᵇ	3.23	Downregulated on Day 4	0.015857
**318**	0.13 ± 0.05ᵃ	0.02 ± 0.008ᵇ	6.50	Downregulated on Day 4	0.000283
**341**	0.10 ± 0.02ᵃ	0.05 ± 0.01ᵇ	2.00	Downregulated on Day 4	0.021799

Protein expression levels are presented as normalized spot volumes (mean ± SE). The ratio represents the fold change (Day 1/Day 4). A ratio >1 indicates higher expression on Day 1 (downregulated on Day 4), whereas a ratio <1 indicates higher expression on Day 4 (upregulated on Day 4). Regulation direction is indicated in the “Regulation (Day 4 vs Day 1)” column.

**Table 3:** Proteins identified in mouse endometrium epithelial cells on Days 1 and 4 of pregnancy.

**Spot No.**	**Protein identity**	**GenBank** **Accession**	**Gene** **symbol**	**Source** **species**	**Theoretical** **Mr/** **pI**	**MALDI-MS** **PMFᵃ**	**MALDI-TOF/** **TOF LIFTᵇ**	**Score/** **threshold** **TOF**	**Score/** **threshold TOF/TOF**	**Tolerance TOF (ppm)**
7	Glutathione S-transferase Mu	gi|6680121	*Gstm2*	*Mus musculus*	25871/7.60	18/87(59)	3(26,47,88)	154/64	161/35	100
16	Creatine kinase U-type, mitochondrial precursor	gi|6753428	*Ckmt1*	*M. musculus*	47373/9.3	16/112(41)	3(30,44,72)	122/64	147/35	100
23	Uncharacterized protein LOC433182	gi|70794816	na	*M. musculus*	47453/6.4	17/100(46)	2(54,88)	116/64	142/35	100
29	Keratin, type I cytoskeletal 19	gi|6680606	*Krt19*	*M. musculus*	44515/5.1	19/134(54)	3(14,32,42)	157/64	88/36	100
33	Keratin, type II cytoskeletal 75	gi|29789317	*Krt75*	*M. musculus*	59932/9.1	14/125(23)	na	65/64	na	100
39	Stress-70 protein, mitochondrial	gi|162461907	*Hspa9*	*M. musculus*	73701/5.7	36/118(54)	4(28, 42, 58, 76)	198/64	204/35	100
46	Protein disulfide isomerase A3 precursor	gi|112293264	*Pdia3*	*M. musculus*	57099/5.8	24/114(55)	2(6,41)	150/64	47/36	100
52	78 kDa glucose-regulated protein precursor	gi|254540166	*Hspa5*	*M. musculus*	68570/5	26/124(38)	2(56,61)	123/64	117/35	100
55	Protein disulfide isomerase precursor	gi|42415475	*Pdia1*	*M. musculus*	57422/4.32	22/152(55)	3(38,83,112)	149/64	174/36	100
56	Protein disulfide isomerase precursor	gi|42415475	*Pdia1*	*M. musculus*	57422/4.62	13/50(36)	3(25,42,53)	117/64	120/35	100
60	Hspd1 protein, partial	gi|76779273	*Hspa1*	*M. musculus*	59559/8.88	17/120(24)	2(10, 38)	93/64	48/35	100
94	14-3-3 protein sigma	gi|3065927	*Sfn*	*M. musculus*	27803/4.6	14/80(52)	3(11, 18, 32)	96/64	49/35	100
116	Sickle tail protein	gi|152061323	*Skt*	*M. musculus*	213874/8.3	37/125(23)	na	96/64	na	100
137	EndoA' cytokeratin (5' end put.); putative	gi|309215	*EndoA* *'*	*M. musculus*	53210/5.3	20/125(36)	3(23,29,76)	86/64	128/34	100
144	Vimentin	gi|55408	*Vim*	*M. musculus*	53746/4.9	23/58(39)	na	140/64	na	100
145	Reticulocalbin 3, EF-hand calcium binding domain, isoform CRA_a	gi|13529539	*Rcn3*	*M. musculus*	39048/4.6	17/135(53)	2(17,23)	114/64	40/35	100
154	mCG17595, isoform CRA_a	gi|148672065	*mCG17595*, isoform CRA_a	*M. musculus*	31866/4.8	65/43(21)	na	65/64	na	100
156	Lamin B2	gi|228591	*Lmnb2*	*M. musculus*	67476/5.3	143/107(37)	3(16,26,38)	143/64	80/36	100
160	Glial fibrillary acidic protein	gi|51066	*Gfap*	*M. musculus*	48494/5.2	na	1(1)	na	44/35	100
179	Prelamin-A/C isoform A precursor	gi|162287370	*Lmna*	*M. musculus*	74478/6	223/93(53)	2(28,68)	223/64	96/34	100
197	Prelamin-A/C isoform A precursor	gi|162287370	*Lmna*	*M. musculus*	74478/6.6	39/95(57)	3(16, 32, 36)	264/64	84/34	100
211	Prelamin-A/C isoform A precursor	gi|162287370	*Lmna*	*M. musculus*	74478/6.6	30/83(47)	1(1)	184/64	38/35	100
222	Spectrin alpha chain, non-erythrocytic 1 isoform 1	gi|115496850	*Sptan1*	*M. musculus*	286025/5.1	28/80(13)	na	65/64	na	100
224	Albumin	gi|26986064	*Alb*	*M. musculus*	20075/6	9/110(57)	4(25,37,45,76)	75/64	137/35	100
229	ATP synthase beta-subunit	gi|2623222	na	*M. musculus*	56344/5	1/108(3)	1(69)	na	69/34	100
236	Unnamed protein product	gi|26341396	na	*M. musculus*	67013/5.4	15/129(31)	4(36,48,96,148)	100/64	346/34	100
246	Glutathione S-transferase Mu 7	gi|113679874	*Gstm7*	*M. musculus*	25864/6.4	17/61(58)	1(46)	125/64	46/35	100
249	Adenylate kinase 2, mitochondrial isoform b	gi|34328230	*Ak2*	*M. musculus*	32661/6	11/66(59)	2(10, 37)	86/64	47/34	100
276	Arginase-1	gi|7106255	*Arg1*	*M. musculus*	34927/6.58	14/79(59)	3(43, 57, 70)	155/64	170/35	100
284	Uncharacterized protein LOC433182	gi|70794816	na	*M. musculus*	47453/6.4	22/135(60)	3(44,80,87)	104/64	211/35	100
287	Uncharacterized protein LOC433182	gi|70794816	na	*M. musculus*	47453/6.4	22/143(62)	3(28,55,76)	127/64	159/34	100
302	Serine (or cysteine) peptidase inhibitor, clade H, member 1, isoform CRA_a	gi|148684430	*Serpinh1*, *Hsp47*	*M. musculus*	45069/9.5	23/97(58)	3(70,71,116)	140/64	258/35	100
314	Fructose-bisphosphate aldolase A isoform 2	gi|6671539	*Aldoa*	*M. musculus*	39787/9.2	15/131(51)	3(10,40,44)	95/64	92/35	100
315	3-ketoacyl-CoA thiolase A, peroxisomal precursor	gi|18700004	*Acaa2*	*M. musculus*	44382/9.7	16/101(49)	2(12,63)	103/64	75/35	100
318	Unnamed protein product	gi|26328539	na	*M. musculus*	82105/10.3	17/99(22)	na	78/64	na	100
341	Heat shock 70 kDa protein 5 (glucose-regulated protein), isoform CRA_b	gi|148676670	*Hspa5*	*M. musculus*	56314/4.9	14/76(34)	2(9, 47)	101/64	57/35	100
350	Atp5b protein	gi|23272966	*Atp5b*	*M. musculus*	56632/5.1	4/114(29)	4(8,61,74,98)	na	241/35	100
357	Vimentin	gi|2078001	*Vim*	*M. musculus*	51590/48	31/119(61)	3(39,52,68)	180/64	164/35	100
385	Vimentin	gi|2078001	*Vim*	*M. musculus*	51590/4.8	26/109(54)	na	143/64	na	100
424	Zinc finger protein mfg2	gi|199139	*Mfg2*	*M. musculus*	48276/10.4	8/61(24)	na	70/64	na	100
484	Heat shock protein 65	gi|51455	*Hsp65*	*M. musculus*	61074/58	19/114/126(6)	2(46,72)	84/64	118/35	100
502	Glutathione reductase 1	gi|148703470	*Gsr*	*M. musculus*	46307/7.95	13/91(50)	na	88/64	na	100
550	Serum albumin precursor	gi|163310765	*Alb*	*M. musculus*	70700/5.7	14/104(29)	4(12, 63, 78, 80)	101/64	231/33	100
555	Short-chain specific acyl-CoA dehydrogenase, mitochondrial precursor	gi|31982522	*Acads*	*M. musculus*	45146/9.4	15/111(40)	3(7,16,17)	75/64	41/35	100
563	Peptidyl-prolyl cis-trans isomerase D	gi|13385854	*Ppid*	*M. musculus*	41116/7.8	12/129(34)	2(21,50)	74/64	71/36	100
573	Acetyl-Coenzyme A acyltransferase 2 (mitochondrial 3-oxoacyl-Coenzyme A thiolase)	gi|20810027	*Acaa2*	*M. musculus*	42288/9.3	20/123(65)	3(51,55,90)	122/64	196/36	100
597	Peroxiredoxin-2	gi|148747558	*Prdx2*	*M. musculus*	23760/7.2	1/89(5)	1(37)	na	37/36	100
603	Cathepsin B	gi|50597	*Ctsb*	*M. musculus*	3046/5.5	1/41(65)	1(65)	na	65/35	100
622	Pyruvate carboxylase, mitochondrial isoform 1	gi|251823980	*Pcx*	*M. musculus*	130491/6.3	32/110(31)	3(16,32,67)	130/64	115/35	100
633	Sickle tail protein isoform b	gi|46358401	*Skt*	*M. musculus*	146438/9.8	21/74(20)	na	75/64	na	100
664	Vimentin V	gi|2078001	*Vim*	*M. musculus*	51590/4.8	25/104(48)	3(5,43,54)	129/64	104/35	100
673	Catalase	gi|442441	*Cat*	*M. musculus*	59982/8.8	14/128(33)	2(31,41)	94/64	72/36	100

MALDI-MS, matrix-assisted laser desorption/ionization mass spectrometer; na, no significant match; PMF, peptide mass fingerprinting.

ᵃ The column refers to the results of the MALDI-MS PMF analysis, i.e., the number of assigned peptides and percent sequence coverage (in parentheses). ᵇ The column refers to the results of the MALDI-MS/MS analysis, i.e., to the number and Mascot scores (in parentheses) of assigned peptides.

### Functional classification of differentially expressed proteins

**Figure 2:**
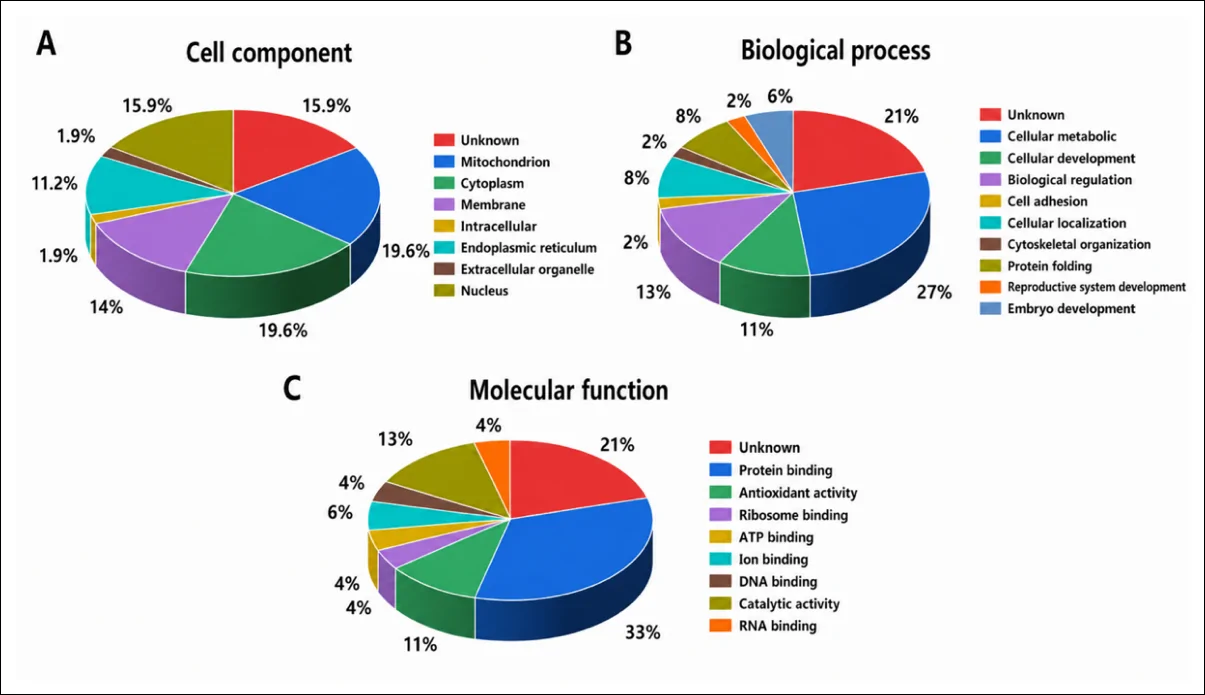
Classification of differentially expressed proteins identified in this study. The identified proteins were annotated using the Gene Ontology database AmiGO 2 and categorized according to (A) subcellular localization, (B) biological processes, and (C) molecular functions.

The 52 identified proteins were functionally annotated for subcellular localization, biological processes, and molecular functions using the AmiGO 2 Gene Ontology database, and the distribution of associated ontological categories is presented in Figure 2. Subcellular localization analysis revealed that the majority of proteins were localized to the cytoplasm (21%) and mitochondria (21%), followed by the membrane (15%), ER (12%), and nucleus (10%). Smaller proportions were detected in intracellular compartments (2%) and extracellular organelles (2%), whereas approximately 17% of the proteins could not be classified. Functional categorization based on biological processes demonstrated that these proteins were primarily associated with cellular metabolism (27%), biological regulation (13%), cellular development (11%), cellular localization (8%), and protein folding (8%). Additional roles included embryonic development (6%), cell adhesion (2%), cytoskeletal organization (2%), and reproductive system development (2%), while 21% of the proteins were not assigned to a specific biological process. Molecular function analysis indicated that most proteins were associated with protein binding (33%), catalytic activity (13%), antioxidant activity (11%), ion binding (6%), ATP binding (4%), ribosome binding (4%), DNA binding (4%), and RNA binding (4%), with approximately 21% remaining functionally uncharacterized. Collectively, these results highlight the diverse molecular pathways involved in endometrial remodeling during early pregnancy (Figure 2). This Gene Ontology analysis was performed for descriptive functional classification only and did not include statistical enrichment testing, multiple-testing correction, or comparison to a background gene set. Notably, several antioxidant proteins (*Gstm2*, *Gstm7*, *Prdx2*, and *Cat*) and ER stress-associated proteins (PDIA3 and HSPA5) exhibited coordinated expression changes, suggesting integrated regulation of oxidative stress and protein-folding stress responses during uterine receptivity.

### Expression of vimentin and *Gstm2* during early pregnancy

**Figure 3:**
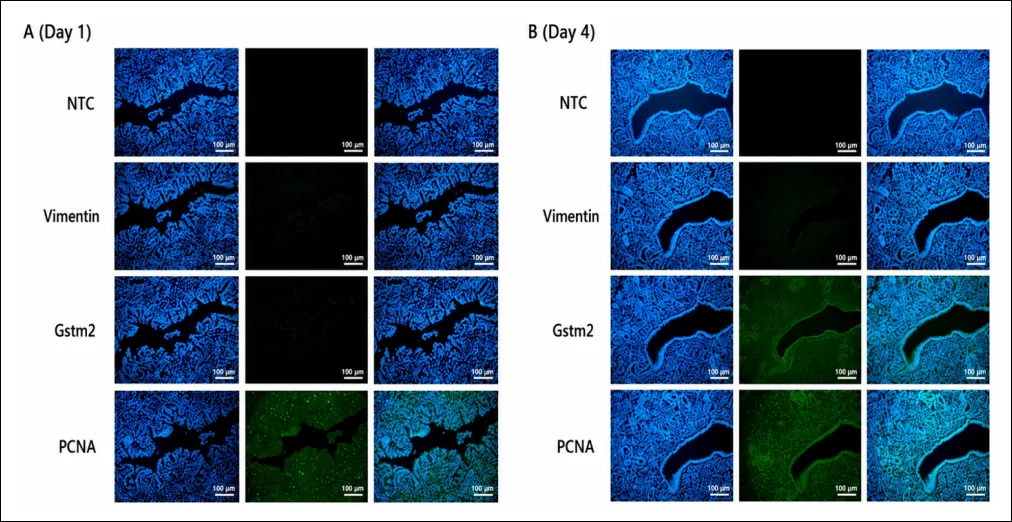
Immunolocalization of vimentin, *Gstm2*, and PCNA (green) in the mouse uterus on Days 1 (A) and 4 (B) of pregnancy. The negative control (NTC) was prepared by replacing the primary antibody with blocking buffer. Nuclei were counterstained with DAPI (blue). Merged fluorescent images of uterine sections were obtained through immunofluorescence staining. Scale bar: 100 μm. Immunofluorescent detection of vimentin, *Gstm2*, and PCNA was performed on uterine cross-sections to visualize epithelial and stromal compartments. Imaging was optimized for the epithelial regions; therefore, stromal vimentin-positive cells may appear faint. Gene expression analysis (quantitative real-time polymerase chain reaction) was performed on isolated epithelial cells to complement immunofluorescence findings.

**Figure 4:**
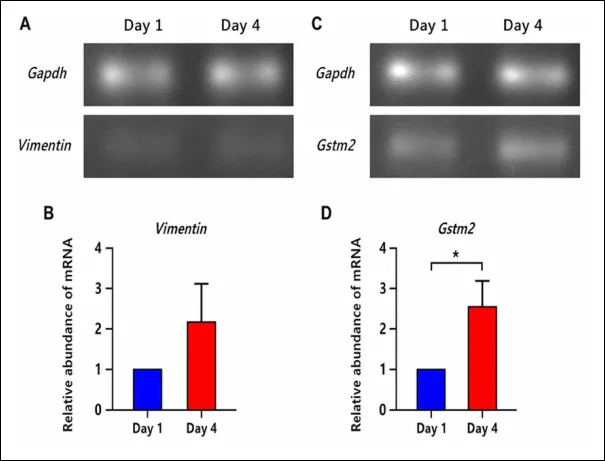
Gene expression in mouse endometrial epithelial cells on Days 1 and 4 of pregnancy. (A and C) Representative agarose gel images showing the expression of *Vimentin* and *Gstm2* following reverse-transcription PCR. (B and D) Relative expression levels of vimentin and *Gstm2* determined by quantitative real-time polymerase chain reaction (qPCR). Glyceraldehyde-3-phosphate dehydrogenase (*GAPDH*) was used as the internal reference for normalization. Data are presented as the mean ± standard error of the mean (SEM) from three independent experiments. Asterisks (*) indicate significant differences in gene expression between Days 1 and 4 (p < 0.05). Note that the qPCR-derived relative transcript abundance may not be fully reflected by the band intensity observed in agarose gel visualization.

Immunofluorescence analysis was performed to determine the localization of vimentin and *Gstm2* in uterine tissue sections collected on Days 1 and 4 of pregnancy (Figure 3). On Day 1, vimentin and *Gstm2* signals were either absent or barely detectable in the luminal and glandular epithelia (Figure 3A). By contrast, on Day 4, low vimentin expression was observed in these epithelial regions, whereas *Gstm2* exhibited strong immunoreactivity (Figure 3B). In addition, PCNA staining was more intense on Day 4 than on Day 1, indicating increased cellular proliferation during the implantation window (Figure 3A and B). To further validate these observations, reverse-transcription PCR and qPCR were performed to assess *Vimentin* and *Gstm2* mRNA expression levels in isolated endometrial epithelial cells. *Vimentin* mRNA expression did not differ significantly between Day 1 and Day 4 (p > 0.05; Figures 4A and B). However, *Gstm2* mRNA levels were significantly higher on Day 4 than on Day 1 (p < 0.05; Figures 4C and D). Although agarose gel electrophoresis (Figure 4A) demonstrated comparable *Vimentin* band intensities between the two time points, qPCR (Figure 4B) indicated a slight increase in *Vimentin* transcript levels on Day 4. The lack of statistical significance may be attributable to biological variability among replicates.

## DISCUSSION

### Endometrial epithelial proteomic remodeling during uterine receptivity

Embryo implantation is a tightly regulated biological process in which the blastocyst attaches to and interacts with the uterine endometrium [21]. The endometrium is a hormonally responsive mucosal tissue lining the uterus that provides structural support, nourishment, and immunological tolerance for the developing conceptus. Transformation of the endometrium into a receptive state requires coordinated regulation of epithelial differentiation, stromal remodeling, immune modulation, and metabolic adaptation [13, 22]. Ovarian steroid hormones, particularly progesterone, regulate adhesion molecules, cytokines, and growth factors that collectively determine uterine receptivity and embryo–maternal communication [23, 24]. In mice, embryo attachment occurs on Day 4 of pregnancy following vaginal plug detection on Day 1 and coincides with luminal closure and epithelial remodeling [11].

In the present study, comparative proteomic profiling of mouse endometrial epithelial cells between Days 1 and 4 of pregnancy identified 80 differentially expressed proteins, of which 52 were successfully characterized. Functional annotation indicated that the identified proteins were primarily associated with biological processes, including cellular metabolism, biological regulation, cytoskeletal organization, protein folding, antioxidant activity, and reproductive system development. Collectively, these findings suggest that the acquisition of uterine receptivity involves coordinated metabolic activation, stress adaptation, and structural stabilization within the epithelial compartment. Notably, this study is among the relatively few 2-DE/MALDI-TOF-based proteomic analyses focused specifically on isolated mouse endometrial epithelial cells and integrates redox/antioxidant pathways, ER stress responses, and cytoskeletal components into a single epithelial-specific dataset. In contrast to whole-endometrium transcriptomic or proteomic studies and uterine fluid proteomics, which reflect composite signals from multiple cellular compartments or secreted proteins, the present epithelial-focused approach enables more precise characterization of cell type–specific molecular changes at the embryo–maternal interface.

Furthermore, building upon previous work by Ni* et al.* [25], our study extends these findings by situating *Gstm2* within a broader proteomic context and reinforcing its biological relevance through combined proteomic identification and dual validation at both protein and transcript levels. In addition, the Gene Ontology analysis in this study was descriptive and did not include formal enrichment testing; therefore, the reported functional categories should be interpreted as general annotations rather than statistically overrepresented pathways.

Among the identified proteins, PDIA3 and HSPA5 (GRP78) are central regulators of endoplasmic reticulum (ER) homeostasis. PDIA3 (ERp57) functions as a protein disulfide isomerase involved in protein folding and ER stress responses [26]. GRP78 is a key ER chaperone participating in unfolded protein response signaling and trophoblast function [26]. Controlled ER stress signaling has been implicated in implantation and placental development, facilitating cellular adaptation during periods of increased biosynthetic demand. The modulation of these proteins in our dataset suggests that epithelial cells undergo adaptive stress responses during the transition to uterine receptivity.

### Antioxidant regulation and the role of *Gstm2*

Redox regulation emerged as another prominent functional category. Successful pregnancy requires balanced oxidative signaling; excessive reactive oxygen species may impair epithelial integrity and embryo–maternal interactions, whereas controlled redox activity supports implantation-associated remodeling [15, 27]. *Gstm2*, a member of the glutathione S-transferase family, plays an important role in cellular antioxidant defense by catalyzing detoxification reactions [25]. Importantly, *Gstm2* was identified alongside multiple antioxidant and stress-associated proteins, including *Gstm7*, *Prdx2*, *Cat*, PDIA3, and HSPA5, suggesting that it functions within a coordinated progesterone-responsive antioxidant network rather than as an isolated factor.

A previous study by *Ni et al.* [25] demonstrated that *Gstm2* expression in the mouse uterine luminal epithelium is regulated by progesterone during the preimplantation period; however, that study focused on hormone-dependent gene regulation rather than global proteomic context. In the present study, *Gstm2* is not proposed as a novel implantation-related factor; rather, our findings extend this prior observation by placing *Gstm2* within a broader epithelial-specific proteomic framework. In contrast to prior single-gene or transcript-level studies, the present work identifies *Gstm2* through unbiased proteomic profiling and demonstrates its coordinated regulation alongside proteins involved in redox homeostasis, ER stress responses, and metabolic adaptation.

*Gstm2* showed consistent upregulation at both the protein and transcript levels during the receptive phase (Day 4), with increased localization in luminal and glandular epithelial compartments. Importantly, this upregulation occurred alongside coordinated changes in proteins associated with redox regulation, ER stress, and metabolic adaptation, suggesting that *Gstm2* may be associated with a broader antioxidant regulatory network during uterine receptivity. These observations are correlative, and further functional studies will be required to define its mechanistic role.

In addition to *Gstm2*, several well-established markers of uterine receptivity have been reported in previous transcriptomic and proteomic studies, including adhesion molecules such as integrins, mucins, and progesterone-regulated genes such as leukemia inhibitory factor (LIF) and HOXA10 [24, 28]. Although these classical markers were not identified in the present dataset, this may reflect methodological differences, including the epithelial-specific focus and the limited proteome coverage inherent to gel-based approaches. Previous omics studies have demonstrated that uterine receptivity involves coordinated regulation of adhesion, immune modulation, and metabolic adaptation pathways [3, 11, 12]. In this context, our findings complement existing literature by highlighting proteins associated with redox regulation and ER stress responses, suggesting that antioxidant defense mechanisms may represent an additional regulatory layer during the implantation window. Furthermore, although other glutathione S-transferase family members, such as *Gstm7,* were detected, *Gstm2* exhibited the most consistent and pronounced upregulation, supporting its potential relevance within this protein family.

Importantly, the temporal overlap between *Gstm2* upregulation on Day 4 and key implantation-associated events, including luminal closure and epithelial remodeling, suggests its involvement in a progesterone-responsive regulatory framework during the establishment of uterine receptivity. Progesterone regulates uterine fluid absorption and lumen closure, processes essential for blastocyst immobilization and implantation [29–31]. Elevated progesterone levels during Days 3 and 4 of pregnancy have been associated with increased uterine *Gstm2* expression [32–34]. Thus, *Gstm2* may be associated with progesterone-regulated pathways, although this relationship was not directly examined in the present study. Together, these observations support the interpretation that *Gstm2* operates within a progesterone-responsive antioxidant network that is temporally aligned with critical implantation events in the uterine epithelium.

### Cytoskeletal organization and extracellular matrix remodeling

In contrast, vimentin expression did not differ significantly between Days 1 and 4 of pregnancy, although immunofluorescence confirmed its localization within stromal and decidual compartments. Vimentin is a major intermediate filament protein that maintains cytoskeletal integrity and structural resilience [35–37]. Cytoskeletal remodeling is critical during decidualization and trophoblast invasion; however, the early preimplantation window evaluated here precedes extensive trophoblast penetration. The relative stability of vimentin expression during this period likely reflects preservation of structural readiness rather than active remodeling.

Developmental studies indicate that trophoblast giant cells begin expressing vimentin around embryonic Day 7.5, coinciding with vascular remodeling and exposure to maternal blood flow [38]. Earlier work demonstrated that trophoblast expansion and formation of maternal blood sinuses occur between embryonic Days 6.5 and 7.5 [39, 40]. Therefore, the absence of significant vimentin modulation between Days 1 and 4 is consistent with the timing of implantation events. Rather than serving as a dynamically regulated marker during early receptivity, vimentin likely provides a stable cytoskeletal scaffold that maintains endometrial structural integrity prior to invasive placentation.

Additional identified proteins, including cathepsin B and HSP47, are associated with extracellular matrix remodeling and collagen processing [41–43]. Extracellular matrix turnover and collagen stabilization are essential components of implantation and placentation. Their presence among differentially expressed proteins further underscores the coordinated structural and functional adaptation occurring within the receptive endometrium. The integration of proteomic profiling with immunolocalization and quantitative gene expression analysis strengthens the reliability of these findings, as multi-level validation enhances biological confidence and reduces the likelihood of false-positive identification [44]. The concordant upregulation of *Gstm2* at both protein and transcript levels supports its biological relevance during the implantation window, consistent with evidence emphasizing the importance of redox homeostasis in uterine receptivity [15, 45]. Meanwhile, stable vimentin expression confirms its role in maintaining baseline cytoskeletal organization during early pregnancy.

### Study limitations, methodological considerations, and future perspectives

Several methodological considerations should be acknowledged. Pooling of epithelial samples was employed to ensure sufficient protein yield and to minimize inter-individual variability, a strategy commonly used in exploratory proteomic studies [46]. However, this approach may obscure biological variation among individual animals. To address this limitation, selected candidate proteins were independently validated using immunofluorescence and quantitative real-time PCR, thereby supporting the reliability of the observed expression patterns.

Although gel-based proteomics provides lower proteome coverage than modern high-resolution LC-MS/MS approaches, 2-DE/MALDI-TOF remains a robust and complementary strategy for resolving protein isoforms and post-translational variants, while enabling reproducible comparative profiling of relatively abundant proteins. In the present study, this approach was particularly suited to the analysis of isolated endometrial epithelial cells, where protein yield is inherently limited, and was aligned with the exploratory objective of capturing major proteomic shifts during the transition to uterine receptivity. Importantly, this platform enables direct visualization of protein expression patterns, which facilitates biological interpretation in a cell type–specific context. Nevertheless, future studies employing high-resolution quantitative LC-MS/MS will be essential for extending proteome coverage and further refining these findings. In this study, its use was further supported by the limited protein yield from isolated epithelial cells and the exploratory objective of identifying major proteomic changes during uterine receptivity.

Furthermore, antibody validation in this study was limited to negative controls and consistency with known expression patterns, and additional validation using orthogonal approaches (e.g., Western blotting or genetic models) would strengthen confidence in protein specificity. Although stringent fold change thresholds were applied to minimize false-positive identifications, future investigations employing quantitative LC-MS/MS platforms combined with targeted functional assays will be necessary to establish causal relationships between candidate proteins and implantation outcomes. In addition, the absence of a formal multiple-testing correction may increase the likelihood of false-positive identifications, as approximately 5% of detected spots are expected to reach statistical significance by chance alone.

Furthermore, several methodological and biological limitations should be considered when interpreting the present findings. First, the use of two-dimensional gel electrophoresis (2-DE) inherently introduces analytical bias, as this technique preferentially detects abundant, soluble proteins within a moderate range of isoelectric points and molecular weights, potentially underrepresenting low-abundance, membrane-associated, or extreme pI/MW proteins. Second, although endometrial epithelial cells were isolated using established protocols, the possibility of minor contamination from stromal or other uterine cell types cannot be completely excluded, which may influence the detected proteomic profile. Third, while Day 4 samples were collected based on established peri-implantation timing following vaginal plug detection, the presence of embryos was not directly confirmed by uterine flushing. Therefore, the observed proteomic changes may primarily reflect maternal hormonal and physiological regulation rather than embryo-derived signaling. Finally, this study was conducted using a single outbred mouse strain (CD-1), and thus, the generalizability of the findings to other strains or species may be limited. Future studies incorporating embryo verification, improved cell type purification, and high-resolution proteomic approaches across multiple genetic backgrounds will further refine the understanding of uterine receptivity mechanisms.

### Translational relevance and integration with contemporary omics studies

Endometrial receptivity is a key determinant of implantation success in assisted reproductive technologies, and impaired receptivity is a major contributor to recurrent implantation failure (RIF) [11, 12, 24]. In recent years, considerable effort has been directed toward identifying reliable molecular biomarkers to improve assessment of the window of implantation and optimize embryo transfer strategies [23, 24]. Although the present study was conducted in a mouse model, several of the identified pathways, including progesterone-associated signaling, redox homeostasis, and epithelial remodeling, are conserved across species [3, 11]. In this context, the upregulation of *Gstm2* and other proteins involved in antioxidant defense and ER stress responses may serve as candidate biomarkers relevant to human endometrial function. Notably, dysregulation of oxidative stress pathways has been associated with impaired receptivity and implantation failure in women [11, 12].

Therefore, the candidate proteins identified in this study may represent components of a broader molecular framework underlying endometrial competence. However, translating these findings into clinical application requires validation in human endometrial samples, particularly in patients with RIF or undergoing assisted reproduction, as well as integration with existing receptivity biomarkers. Although *Gstm2* was identified as a significantly altered protein in this study, it should not be considered a standalone marker of endometrial receptivity. Rather, it represents a potential candidate biomarker within a complex molecular network, as evidenced by the identification of multiple proteins associated with this process. Due to the exploratory nature of this study, validation was limited to a subset of candidates; future studies should systematically validate additional key candidates (e.g., PDIA3, HSPA5/GRP78, HSP47, and antioxidant-related proteins) using targeted and functional approaches.

Recent advances in transcriptomic and multi-omics approaches, including single-cell RNA sequencing and uterine fluid proteomics, have significantly expanded our understanding of endometrial receptivity by revealing cell type–specific differentiation trajectories, immune regulation, and hormone-responsive signaling pathways during the implantation window [3, 11, 12]. In particular, single-cell transcriptomic studies have highlighted dynamic epithelial remodeling and coordinated interactions between epithelial, stromal, and immune compartments, while human studies in RIF have identified dysregulation of pathways related to cellular stress, metabolism, and hormonal responsiveness [5, 12]. However, most of these studies are based on transcript-level data or whole-tissue analyses, which may not fully capture protein-level changes or cell type–specific molecular dynamics at the epithelial interface.

In this context, the present study complements existing literature by providing an epithelial-specific proteomic perspective, revealing coordinated changes in proteins associated with redox regulation and ER stress responses during the transition to uterine receptivity. Notably, the identification of antioxidant proteins (*Gstm2*, *Gstm7*, *Prdx2*, and *Cat*) together with ER chaperones (PDIA3 and HSPA5) suggests that redox homeostasis and protein folding capacity are jointly modulated in epithelial cells, representing an additional layer of regulation that may not be fully resolved by transcriptomic approaches alone. Within this framework, *Gstm2* is not interpreted as a standalone regulatory factor but rather as a candidate protein whose upregulation is associated with broader epithelial stress-adaptation processes during the receptive phase. These findings are consistent with accumulating evidence linking oxidative stress regulation to endometrial function and implantation success [11, 12], while emphasizing the importance of integrating protein-level data to refine current models of uterine receptivity.

## CONCLUSION

This study demonstrated that mouse endometrial epithelial cells undergo substantial proteomic remodeling during the transition from Day 1 to Day 4 of pregnancy, corresponding to the establishment of uterine receptivity. Comparative proteomic analysis identified 80 differentially expressed protein spots, of which 52 proteins were successfully characterized. Functional classification revealed coordinated regulation of proteins associated with cellular metabolism, antioxidant defense, ER stress responses, cytoskeletal organization, protein folding, and reproductive system development. Notably, *Gstm2* exhibited consistent upregulation at both the protein and transcript levels during the receptive phase, whereas vimentin expression remained relatively stable, suggesting distinct functional roles in epithelial adaptation and structural maintenance during early pregnancy.

The findings provide important insights into the molecular events occurring at the embryo–maternal interface and highlight the potential contribution of redox homeostasis and stress adaptation pathways to successful implantation. The coordinated modulation of antioxidant-related proteins (*Gstm2*, *Gstm7*, *Prdx2*, and *Cat*) together with ER-associated proteins (PDIA3 and HSPA5) suggests that the maintenance of oxidative balance and protein-folding capacity may be integral components of epithelial preparation for embryo attachment. These observations contribute to the growing body of evidence that uterine receptivity is governed by interconnected molecular networks rather than individual regulatory factors.

A major strength of this study is the use of isolated endometrial epithelial cells, which enabled cell type–specific characterization of proteomic changes that may be obscured in whole-tissue analyses. Furthermore, integration of proteomic profiling with immunofluorescence and quantitative real-time PCR validation increased confidence in the biological relevance of the identified candidates. Nevertheless, several limitations should be considered, including the study's exploratory nature, the use of pooled samples, the limited proteome coverage associated with gel-based proteomics, the absence of embryo verification via uterine flushing, and the lack of functional validation experiments to establish causal relationships between candidate proteins and implantation outcomes.

Future investigations should employ high-resolution quantitative proteomic platforms, improved epithelial cell purification strategies, and functional approaches such as gene knockdown, overexpression, or conditional knockout models to clarify the mechanistic roles of candidate proteins during implantation. Validation in additional mouse strains and human endometrial samples, particularly from women experiencing RIF, will be essential to determine the translational relevance of these findings.

Overall, the present study provides a comprehensive epithelial-specific proteomic profile of the mouse endometrium during early pregnancy and identifies coordinated changes in antioxidant, stress-response, metabolic, and structural pathways associated with uterine receptivity. These findings establish a valuable molecular framework for future studies to elucidate implantation mechanisms and identify biomarkers or therapeutic targets relevant to reproductive success.

## DATA AVAILABILITY

The supplementary materials include representative 2-DE gel images, protein spot quantification data, protein identification results, MALDI-TOF/MS spectra, peptide mass fingerprinting data, peak lists, Mascot search outputs and parameters, Gene Ontology annotation results, immunofluorescence and quantitative real-time PCR data, and Melanie 7 software quantification datasets. The raw and processed proteomics data are currently being prepared for deposition in the PRIDE repository through the ProteomeXchange Consortium. The accession number and reviewer access credentials will be provided upon completion of the deposition process and will be included in the published version of the article. Additional data supporting the conclusions of this study are available from the corresponding author upon reasonable request.

## GENERATIVE AI DECLARATION

During the preparation of this manuscript, the authors used ChatGPT (OpenAI) solely to improve the English language, grammar, sentence structure, and overall readability of the manuscript. All AI-assisted suggestions were carefully reviewed, verified, and revised by the authors. The authors take full responsibility for the accuracy, originality, and integrity of the manuscript. Generative AI was not used to generate scientific content, analyze or interpret data, or draw scientific conclusions. 

## AUTHORS’ CONTRIBUTIONS

JJ and WI contributed equally to this study. JJ: Conceptualization, data curation, formal analysis, methodology, visualization, writing – original draft, and writing – review and editing. WI: Conceptualization, data curation, formal analysis, methodology, writing – original draft, and writing – review and editing. AT: Investigation and writing – review and editing. SW: Conceptualization and methodology. CC: Methodology and formal analysis. YL: Data curation and writing – review and editing. TM: Data curation, formal analysis, and writing – review and editing. SH: Conceptualization and methodology. PT: Conceptualization, methodology, project administration, supervision, and writing – review and editing. All authors have read and approved the final manuscript.
